# Changes in and asymmetry of the proteome in the human fetal frontal lobe during early development

**DOI:** 10.1038/s42003-022-04003-6

**Published:** 2022-09-29

**Authors:** Xiaotian Zhao, Wenjia Liang, Wenjun Wang, Hailan Liu, Xiaolei Zhang, Chengxin Liu, Caiting Zhu, Baoxia Cui, Yuchun Tang, Shuwei Liu

**Affiliations:** 1 Department of Anatomy and Neurobiology, Research Center for Sectional and Imaging Anatomy, Shandong Provincial Key Laboratory of Mental Disorder, Shandong Key Laboratory of Digital Human and Clinical Anatomy, School of Basic Medical Sciences, Cheeloo College of Medicine, Shandong University, Jinan, 250012 Shandong China; 2 Institute of Brain and Brain-Inspired Science, Shandong University, Jinan, 250012 Shandong China; 3 Department of Obstetrics and Gynecology, Qilu Hospital of Shandong University, Cheeloo College of Medicine, Shandong University, Jinan, 250012 China

**Keywords:** Developmental neurogenesis, Brain, Neuronal development, Protein databases, Developmental neurogenesis

## Abstract

Inherent hemispheric asymmetry is important for cognition, language and other functions. Describing normal brain and asymmetry development during early development will improve our understanding of how different hemispheres prioritize specific functions, which is currently unknown. Here, we analysed developmental changes in and asymmetry of the proteome in the bilateral frontal lobes of three foetal specimens in the late first trimester of pregnancy. We found that during this period, the difference in expression between gestational weeks (GWs) increased, and the difference in asymmetric expression decreased. Changes in the patterns of protein expression differed in the bilateral frontal lobes. Our results show that brain asymmetry can be observed in early development. These findings can guide researchers in further investigations of the mechanisms of brain asymmetry. We propose that both sides of the brain should be analysed separately in future multiomics and human brain mapping studies.

## Abstract (web summary)

Proteomic analysis of human early fetal brain tissue is undertaken to investigate bilateral developmental changes of protein expression and left-right asymmetries of protein expression.

## Introduction

Asymmetry, a basic feature of the human brain and other vertebrate brains, has been a concern of neuroscientists since Marc Dax and Gustave Dax argued that the left hemisphere plays a leading role in speech in the 19th century^1^. At the population level, differences between the left and right hemispheres exist at almost all levels of the brain, including anatomical structure^2^, brain region connectivity^3^, and function^4^. Brain asymmetry is critical for the maintenance of normal physiological functions in human, and changes in inherent brain asymmetry have been observed in ageing individuals^5^, in people with neurodegenerative diseases^6^ and in individuals with mental disorders^7^.

Brain structure asymmetry has been observed across the lifespan, including during early development before birth^8,9^. Fetal choroid plexus asymmetry can be observed in human foetuses from GWs 11 to 13^10^. It was also found that during this period, the movement of the right arm of the fetus is greater than that of the left arm^11,12^. Because the prenatal brain is less affected by habits and environmental factors, the lateralization of brain structure and motor behavior observed in the first three months of pregnancy is thought to be due to asymmetric genetic-developmental processes^13^.

Genes that are differentially expressed between the left and right hemispheres have also been identified in previous studies^14^. The developmental mechanism of brain lateralization from the middle stage of fetal development to adulthood has been explored. Recent genome-wide association studies (GWAS) have identified genetic loci that show strong associations with regional brain asymmetry^15^. Many genetic variants have been reported to be associated with adult human brain asymmetry at the genome level^16–18^. However, how brain asymmetry is established in the early stage of fetal development is not well understood.

Prenatal transcriptome data have revealed the widespread changes that occur during fetal development^19,20^, and a series of RNA sequencing studies have revealed the differential expression of genes between left and right hemisphere samples from embryos^14,21–24^. However, when protein data was integrated with RNA-seq data, it was found that the difference in protein abundance between brain regions was generally higher than the difference in RNA levels^25^. Furthermore, a wide spectrum of genetic variants associated with diseases is often found at the proteome level. Differences in the expression of many proteins reflect the changes in cellular components and functions, and proteomic data provide direct information about the composition and functional states of proteins. A series of studies in animal models have confirmed the existence of asymmetrical behavior and lateral asymmetry in the left and right hemispheres, including using differential proteomics to assess the hippocampal laterality in healthy rodents^26–28^. Although animal model research helps us to understand the asymmetrical expression of brain proteins, the proteomic changes observed with human brain asymmetry still need to be further elucidated in human specimens. However, given the limitation of study samples and technical limitations, these changes at the protein level remain unclear.

In addition to prenatal brain asymmetry, fetal brain development needs to be urgently studied since many psychiatric disorders in children and adults have been reported to originate before birth^29,30^. In recent years, researchers have extensively studied the development of the human cerebral cortex and the emergence of distinct neuronal lineages at the cellular level^19,31^. Despite this research, changes in the early fetal brain at the proteome level are not fully understood.

**Fig. 1:**
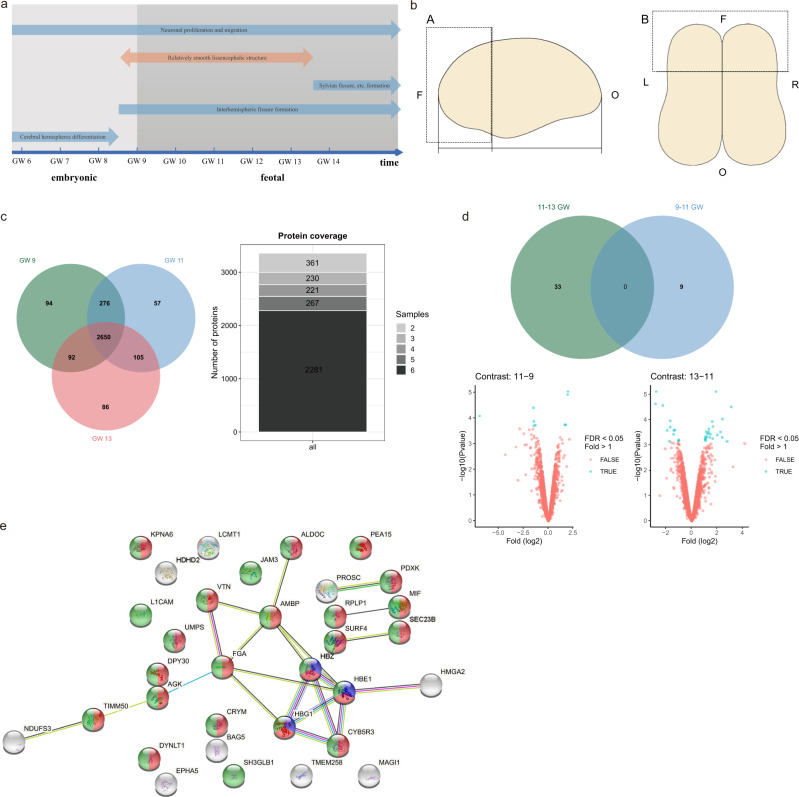
Proteins identified in the fetal frontal lobes at different gestational weeks. **a** The time course of brain development. **b** Dissection of the human fetal frontal lobe. (A) A lateral view of the left hemisphere of the human fetal brain. One-third of the occipitofrontal diameter close to the frontal pole (F) as the frontal lobe is dissected. O, occipital pole. (B) A top view of the human fetal brain. Tissues were dissected from the left (L) and right (R) frontal lobes. **c** The protein set identified in all three samples contained 2650 proteins, and the core protein set identified in all six tissues contained 2281 proteins. **d** Volcano map of core proteins that showed significant differences in abundance between gestational weeks 9–11 and gestational weeks 11–13 (FDR < 0.05, log2Fold > 1). **e** Visualization of the PPI network of proteins showing differential expression between gestational weeks 11 and 13. The PPI network has significantly more interactions than expected (PPI enrichment *p* value: 7.68e-09). The nodes represent proteins, and the proteins enriched in different biological processes are indicated by the different colors; the proteins enriched in the hydrogen peroxide catabolic process are shown in purple (count = 3, FDR = 0.0405), those enriched in transport are shown in red (count = 21, FDR = 0.0027), and those enriched in localization are shown in green (count = 24, FDR = 0.0025). The edges represent the predicted interactions according to STRING. These interactions include known interactions from curated databases (light blue), experimentally determined interactions (magenta), and interactions identified by gene neighborhood (green), gene fusion (red), gene co-occurrences (blue), text mining (yellow), co-expression (black), and protein homology (purple). The whole human genome was used as the statistical background for the functional enrichments.

To explore the genetic-developmental mechanisms underlying typical brain asymmetry, we focused on the frontal lobes, which have been widely reported to exhibit structural asymmetry^32^. We analyzed asymmetrically expressed proteins (AEPs) and observed changes in an early stage of fetal development (GWs 9–13), a critical period for neuroblast proliferation and migration. During this period, the surface of the brain is a smooth lissencephalic structure, as the brain has not yet evolved its many fissures and folds^33^ (Fig. 1a). Our results show that brain asymmetry can be observed in the early stage of fetal development. An important basis for human brain asymmetry is the difference in protein expression before birth, which develops before functional asymmetry. Our study will help identify more sophisticated differences in the normal asymmetric development of the human brain.

## Results

### Proteomes of fetal frontal lobes in GW 9, 11, and 13

To investigate the protein compositions of the frontal lobe in early fetal development, we prepared tissue homogenates from the brains of human fetuses aborted at GWs 9, 11, and 13 (Fig. 1b). Due to different evolutionary processes and the presence of isoforms, some proteins are so similar that individual proteins cannot be distinguished based on their peptide content. To avoid overcounting identifications at the protein level and make quantitative information explicit, in MaxQuant, identifications and quantifications are reported at the group level^34^. ‘Protein’ is used to indicate both individual proteins and protein groups for the sake of simplification. The global false discovery rate (FDR) for peptide and protein identification is set at 0.01. The numbers of proteins in the fetal frontal lobe included in the analyses after filtering were 3112 in GW 9, 3088 in GW 11, and 2933 in GW 13. The complete results from the protein identification in each GW are displayed in Supplementary Data 1. A total of 2650 proteins were identified in all three GWs, as shown in the Venn diagram (Fig. 1c). Although >2700 proteins were detected in the bilateral frontal lobes at different GWs, a subset of proteins (2281) was detected in all tissues (Fig. 1c). This core subset may be less affected by individual differences and may play a continuous role in human brain development. GO enrichment analysis revealed that the core proteins were enriched in the biological processes (FDR < 0.01, Fisher exact test, Supplementary Fig. 1 and Supplementary Data 2) mRNA catabolic process, RNA catabolic process, RNA splicing, and mRNA splicing, which occur widely throughout the development process.

### Differentially expressed proteins (DEPs) between different GWs

To quantitatively compare the protein expression differences in tissues from different samples, we normalized the core protein data with the log2 normalization method. DEPs between samples from different GWs were identified by limma with FDR control 0.05 using the log2 normalized core protein subset. We identified nine DEPs (out of 2281 proteins total (0.39%); four upregulated and five downregulated) between GW 9 and GW 11 and DEPs 33 (1.45%, 21 upregulated and 12 downregulated) between GW 11 and GW 13 (Fig. 1d and Supplementary Data 3 and 4). With development, the number of DEPs increased, consistent with previous findings that the expression of most protein-coding genes changes over prenatal development^35,36^. Among the DEPs, no proteins showed significant expression changes in two time periods (Fig. 1d). DEPs between GW 9 and GW 11 have no significant protein-protein interaction, whereas significant protein-protein interaction (PPI enrichment *p* value: 7.68e-09) was detected between GW 11 and GW 13 (Fig. 1e and Supplementary Data 4). These DEPs between GW 11 and GW 13 were significantly involved in hydrogen peroxide catabolic process (count = 3, FDR = 0.0405; purple), transport (count = 21, FDR = 0.0027; red) and localization (count = 24, FDR = 0.0025; green).

We identified multiple DEPs that play a role in neuronal development and differentiation. In the comparison of GW 11 with GW 9, two proteins associated with neurogenesis were significantly changed as annotated by the UniProt keyword database, and these included alpha-internexin (INA; log2FC = 1.73, FDR = 0.050), which showed upregulated expression, and NDRG2 (NDRG2; log2FC = −1.45, FDR = 0.050), which showed downregulated expression. The most downregulated protein was neuroblast differentiation-associated protein AHNAK (AHNAK; log2FC = −6.88, FDR = 0.048), which may be needed for neuronal cell differentiation^37^, whereas the most upregulated protein was cytochrome c (CYCS; log2FC = 2.01, FDR = 0.014), which is regulated by neuronal activity^38^.

The comparison between the samples from GW 13 and the samples from GW 11 identified three proteins associated with neurogenesis, and their expression increased with an increase in the gestational age. These three proteins were ephrin type-A receptor 5 (EPHA5; log2FC = 2.25, FDR = 0.041), which reportedly functions as an axon guidance molecule during development^39^; neural cell adhesion molecule L1 (L1CAM; log2FC = 1.15, FDR = 0.041), which is related to neuronal migration and axonal growth^40^; and dynein light chain Tctex-type 1 (DYNLT1; log2FC = 1.25, FDR = 0.043), which is related to axon formation^41^. The most upregulated protein was ketimine reductase mu-crystallin (CRYM; log2FC = 3.09, FDR = 0.014), and the most downregulated protein was protein AMBP (AMBP; log2FC = −2.83, FDR = 0.014).

### Proteomes of left and right fetal frontal lobes

**Fig. 2:**
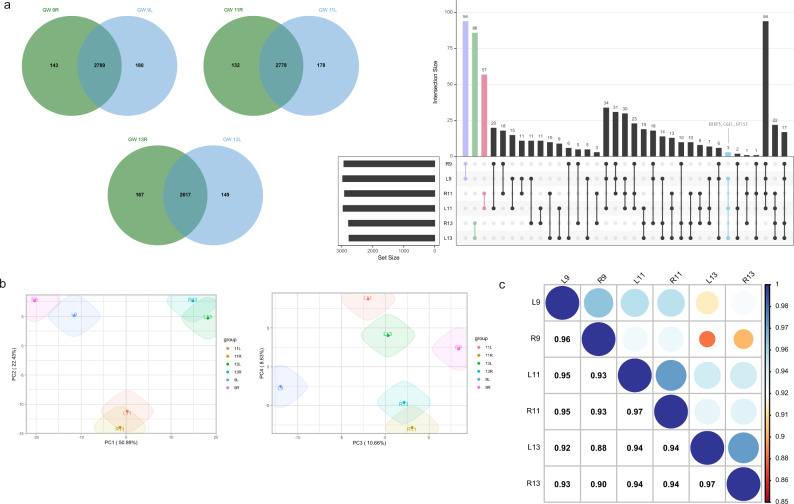
Proteomes of the left and right fetal frontal lobes. **a** Overlapping proteins in the bilateral frontal lobes at each gestational week. An UpSetR diagram was created to visualize the proteins identified in only one gestational week. Three proteins only detected in the left side of the frontal lobe are listed in the figure. **b** PCA plots of PC1 versus PC2, and PC3 versus PC4. The first four principal components account for 92.61% of the total data variance, and the individual contributions of PC1, PC2, PC3, and PC4 are 50.89%, 22.43%, 10.66% and 8.63%, respectively. **c** Pearson correlation analysis of samples from different gestational weeks.

Proteome analysis of the frontal lobe at GW 9 yielded 2969 proteins that were expressed in the left frontal lobe, and 2932 that were expressed in the right frontal lobe. There were 2789 proteins in the bilateral frontal lobes at GW 9. Further, there were 2956 proteins expressed in the left lobe, and 2910 in the right lobe at GW 11, 2778 of which were shared. At GW 13, 2766 proteins were expressed in the left lobe, and 2784 proteins were expressed in the right lobe, 2617 of which were shared (Fig. 2a). There were 2886 proteins per frontal lobe on average. Six data sets from different GWs were subjected to principal component analysis (PCA) and Pearson correlation analysis to reduce the dimensionality of the array data and visualize the sample grouping. Samples from different GWs could be separated, indicating that the differences between hemispheres were more subtle than the differences between GWs (Fig. 2b, c). At the same time, there were differences in protein expression between the left and right frontal lobes in the fetal samples. We used the encircle function of the ggalt package to automatically enclose points in a polygon with the default parameters. The separation of the left and right frontal lobes at 11 and 13 GW was lower than that at 9 GW, that is, the asymmetry degree of the frontal lobe proteome at 11 and 13 GW was generally reduced. PC1 clearly differentiated the samples based on their developmental stages and sides (Fig. 2b).

### AEPs in fetal frontal lobes

**Fig. 3:**
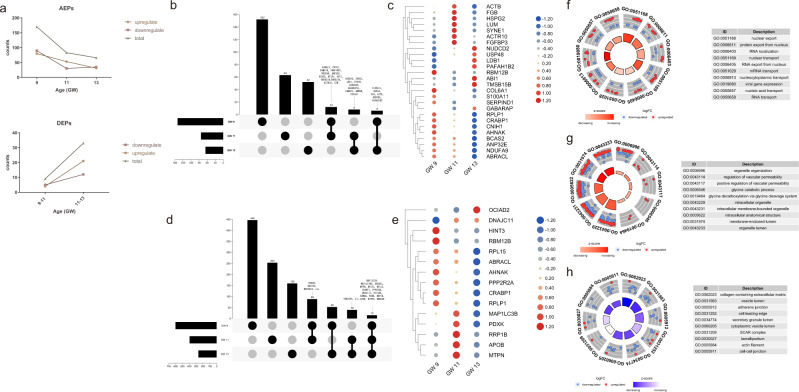
AEPs between the left and right frontal lobes. **a** The number of DEPs increased while the number of AEPs decreased from GW 9 to GW 13. **b** Twenty-six (12 + 8 + 6) core AEPs with twofold changes were shared between two different GWs. **c** Heatmap showing differences in protein expression (fold change) in core AEPs at the three GWs. Values were scaled and clustered by row. **d** Fifteen proteins asymmetrically expressed with a fold change of at least 1.5 were shared between all three GWs. Some noticeable proteins are marked. **e** Heatmap showing differences in protein expression (fold change) in the 15 proteins asymmetrically expressed with a fold change of at least 1.5 shared among all three GWs. Values were scaled and clustered by row. **f**–**h** GO terms enriched by AEPs at GW 9 (**f**), GW 11 (**g**) and GW 13 (**h**). The blue dots represent proteins that were highly expressed on the right side of the frontal lobe, and the red dots represent proteins that were highly expressed on the left side. For a complete list of enriched GO terms, see Supplementary Data 8. AEPs asymmetrically expressed proteins, DEPs differentially expressed proteins, GW gestational week.

In the asymmetric analysis, we conducted pairwise comparisons of samples from the left and right frontal lobes in each GW. AEPs were defined as those detectable in both left and right frontal lobes, with a difference in expression more than twofold. At GW 9, 170 AEPs were identified; among these, 80 AEPs were highly expressed in the left frontal lobe, and 90 AEPs were highly expressed in the right frontal lobe. At GW 11, 83 proteins were asymmetrically expressed, and these included 53 with higher expression on the left side and 30 with higher expression on the right side. At GW 13, 66 AEPs were identified, with 31 being expressed more on the left side and 35 being expressed more on the right side (Supplementary Data 5). Interestingly, unlike the gradual increase in the number of DEPs during development, the number of AEPs between the left and right frontal lobes decreased with increasing gestational age (Fig. 3a). In previous studies, asymmetric expression of some genes was found to become less pronounced with increasing gestational age^14^. We hypothesize that this result is consistent with previous reports of complex changes in brain asymmetry in fetuses and infants^42^.

**Fig. 4:**
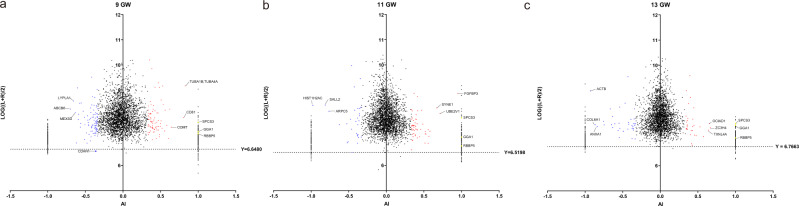
Proteins detected on only one side of the fetal frontal lobe. **a**–**c** Scatter plots showing the average log-normalized LFQ intensity plotted against the asymmetry index ((L−R)/(L+R)) at GWs 9 (**a**), 11 (**b**), and 13 (**c**). Asymmetrically expressed proteins (fold change >2) are represented by red and blue dots. Proteins that showed the three highest levels of asymmetric expression are marked. Three proteins showing unilateral expression at all three GWs are represented by yellow triangles. The horizontal line represents the 1st percentile of the bilaterally averaged intensities of proteins that can be detected. Codanin-1 (CDAN1) is marked due to a lack of sufficient intensity. GWs gestational weeks.

However, the low expression intensity may have resulted in asymmetrical expression by chance and thus does not provide very convincing evidence. To reduce screening errors due to some proteins exhibiting a low label-free quantification (LFQ) intensity on both sides of the frontal lobe, we applied a filter. We defined the asymmetry index (AI) as AI = (L − R)/(L + R). The AI value for proteins only expressed on the left side would equal 1, and that for proteins only expressed on the right side would equal −1. Scatter plots were plotted based on the AI and the average bilateral expression (calculated as log ((L + R)/2)) of each protein to identify unilaterally expressed proteins (Fig. 4a–c, Supplementary Fig. 2). We used the 1st percentile of the protein expression intensity as a credible screening standard; only Codanin-1 (CDAN1) detected in GW 9 did not exhibit sufficient intensity. This protein is displayed in red in Supplementary Data 5 and was not included in the further analysis of AEPs.

We did not identify any AEPs that showed a twofold change in expression at all three GWs, but 26 proteins were asymmetrically expressed at two different GWs (Fig. 3b, c and Supplementary Data 6). These proteins included cytoskeleton-associated proteins, i.e., actin, cytoplasmic 1 (ACTB), actin-related protein 10 (ACTR10), abl interactor 1 (ABI1), thymosin beta-15B (TMSB15B), and gamma-aminobutyric acid receptor-associated protein (GABARAP). Recent studies have suggested that cytoskeleton-related genes are related to brain structure asymmetry^15^. Among those AEPs with twofold change, 60 S acidic ribosomal protein P1 (RPLP1) was also differentially expressed at GW 11–13, and neuroblast differentiation-associated protein AHNAK (AHNAK) was differentially expressed at GW 9 to 11. In addition, NADH dehydrogenase [ubiquinone] 1 alpha subcomplex subunit 9 (NDUFA9) was found to be associated with asymmetry and is known as an accessory subunit of the mitochondrial membrane respiratory chain NADH dehydrogenase (Complex I).

Using a fold change of at least 1.5 as the screening criterion, 15 proteins were found to be differentially expressed at all three GWs (Fig. 3d, e and Supplementary Data 7). RPLP1 was asymmetrically expressed (with a bias toward the left side compared with the right side) at three GWs, whereas myotrophin (MTPN), dnaJ homolog subfamily C member 11 (DNAJC11), cellular retinoic acid-binding protein 1 (CRABP1), OCIA domain-containing protein 2 (OCIAD2), pyridoxal kinase (PDXK) and AHNAK were underexpressed on the left side. Notably, NADH dehydrogenase [ubiquinone] 1 alpha subcomplex subunit 9 and 10 (NDUFA9 and NDUFA10) and microtubule-associated proteins, i.e., tubulin beta-8 chain (TUBB8) and gamma-tubulin complex component 3 (TUBGCP3), showed a 1.5-fold change and asymmetrical expression at two different GWs (Fig. 3d).

GO enrichment analysis was performed to further elucidate the AEPs between the left and right frontal lobes at each GW. Figure 3f–h show the top 10 significantly enriched GO terms in the three categories. AEPs at GW 9 were enriched in biological processes related to nuclear export, RNA localization, RNA transport and mRNA transport (Fig. 3f and Supplementary Data 8). The upregulated proteins on the left side at GW 11 were associated with organelle organization, regulation of vascular permeability and glycine catabolic process (Fig. 3g and Supplementary Data 8). We observed that the AEPs at GW 13 were mainly enriched in terms related to the collagen-containing extracellular matrix, vesicle lumen, adhesion and binding. Most proteins with significant GO enrichment were more highly expressed on the right side than on the left side at GW 13 (Fig. 3h and Supplementary Data 8).

### Proteins detected on only one side of the fetal frontal lobe

After excluding AEPs with expression differences of more than twofold changes, some proteins showed zero intensity in unilateral samples, which may be due to the low peptide signal used for protein quantification, resulting in no peak area integral quantification value. We suggest that proteins with zero detected intensity in the unilateral frontal lobes may also be related to the inherent asymmetry of the brain. For simplicity, we defined these proteins as having unilateral expression. We also used the 1st percentile of the protein expression intensity as a credible screening standard to reduce screening errors caused by a low LFQ intensity. At GW 9, 294 proteins were detected on only one side, at GW 11, 282 proteins were unilaterally expressed, and at GW 13, 289 unilaterally expressed proteins were identified (Supplementary Data 9). Among the unilaterally expressed proteins, roundabout homolog 2 (ROBO2) was unilaterally expressed at GW 9 and 11, and tubulin alpha-1B chain (TUBA1B) was a unilaterally expressed protein at GW 11 and 13. GWASs have revealed that ROBO2 and TUBA1B are significantly associated with handedness and cortical surface area asymmetry^43^. It is noteworthy that we identified three proteins that were detected on only the left frontal lobe at all three GWs (Fig. 4a–c). These three proteins are retinoblastoma-binding protein 5 (RBBP5), ADP-ribosylation factor-binding protein GGA1 (GGA1) and signal peptidase complex subunit 3 (SPCS3). Using UniProtKB keywords, we identified GGA1 and RBBP5 associated with alternative splicing, and SPCS3 participated in the molecular process of peptidase activity. No significant interaction was found between the three proteins expressed only in the left frontal lobe. However, we believe that the interaction between these proteins has not been sufficiently studied.

### Asymmetry of protein changes in the bilateral frontal lobes during development

**Fig. 5:**
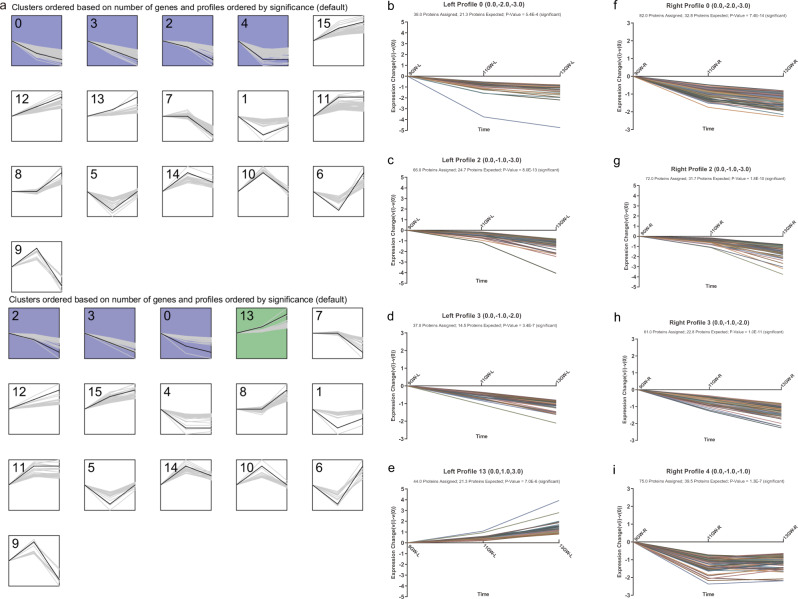
Short time-series expression miner (STEM) analysis of proteins in the bilateral frontal lobes. **a** Results of the STEM analysis of core proteins in the left and right frontal lobes. The upper left corner represents the profile ID. The black line displays the model expression of each profile. The gray lines represent all single gene expression profiles. The *x* axis represents time: 9, 11, and 13 GW. Significantly enriched clusters are indicated by different colors (*p* < 0.05). No significant difference was found in the expression of clusters labeled white. **b** The expression of proteins in cluster 0 on the left side (38 assigned) gradually decreased, and the rate of downregulation decreased with increasing age. **c** The expression of proteins in cluster 2 on the left side (66 assigned) gradually decreased, and the rate of downregulation increased with increasing age. **d** The expression of proteins in cluster 3 on the left side (37 assigned) gradually decreased with increasing age. **e** The expression of proteins in cluster 13 on the left side (44 assigned) gradually increased, and the rate of upregulation increased with increasing age. **f** The expression of proteins in cluster 0 on the right side (82 assigned) gradually decreased, and the rate of downregulation decreased with increasing age. **g** The expression of proteins in cluster 2 on the right side (72 assigned) gradually decreased, and the rate of downregulation increased with increasing age. **h** The expression of proteins in cluster 3 on the right side (61 assigned) gradually decreased with increasing age. **i** The expression of proteins in cluster 4 on the right side (75 assigned) gradually decreased from GW 9 to 11, and almost no change was found between GW 11 and 13. GW gestational week.

To further understand the effect of development on frontal lobe asymmetry, we focused on the expression profiles of the 2281 core proteins, which were clustered into 16 profiles (Fig. 5a) using short time-series expression miner (STEM). Among the 16 expression profiles, five were statistically significant; Supplementary Data 10 shows the proteins belonging to these profiles. We found that the expression patterns of the bilateral frontal lobes were significantly clustered in profiles 0, 2, and 3. The expression of proteins in profiles 0, 2 and 3 decreased with age, but the rate of increase varied. The expression of profile 0 proteins gradually decreased, and the rate of downregulation decreased (Fig. 5b, f). The expression of proteins in profile 2 appeared to decrease faster with increases in the gestational age (Fig. 5c, g), and the proteins in profile 3 showed a significant linear correlation (Fig. 5d, h). Notably, the expression of 44 proteins in profile 13 was positively correlated with age (Fig. 5e), and these proteins were only significantly clustered in the left frontal lobe. Moreover, 75 proteins in profile 4 that were only significantly expressed in the right frontal lobe showed a gradual decrease in expression from GW 9 to 11, and almost no change was found between GW 11 and 13 (Fig. 5i). Functional annotation clustering revealed that the proteins significantly clustered in profile 4 were predominantly involved in formation of the cytoplasmic translation initiation complex (FDR = 0.019) and focal adhesion (FDR = 0.0069; Supplementary Data 11). There may be subtle differences in the changes in the patterns of protein expression in the bilateral frontal lobes during development. This finding confirms the existence of differences in the patterns of protein asymmetrical expression during brain development^22^.

## Discussion

In this study, we investigated the development of the human fetal frontal lobes from GW 9 to GW 13 and observed the difference in protein expression between the left and right frontal lobes. From GW 9 to GW 13, the human brain is a smooth ‘lissencephalic’ structure with no fissures except the cerebral longitudinal fissure; the Sylvian and cingulate fissures begin to appear at GW 14^44^ (Fig. 1a). The changes that occur during this short period, at which point brain morphology has been established but there is no evident cortical folding, are worthy of attention. However, few studies have focused on this period. We found that during this period, the number of DEPs increased, while the number of AEPs decreased. Previous studies have shown that many proteins are expressed during fetal development, but not in normal adult tissues^45^. Transcriptome analysis has revealed found a sharp decrease in regional differences during late fetal development^19^. Moreover, more protein-coding genes exhibit significant changes before birth compared to after birth^35^. At the same time, the difference in protein expression between the left and right frontal lobes may be very difficult to detect in adults with normal physiological function^46^. Our results show that the innate asymmetrical pattern of the brain changes and becomes less obvious with age between GW 9 to 13. We speculate that the proteome of the brain undergoes marked changes during prenatal development, both with developmental changes and due to inherent asymmetry, especially during the late first trimester.

It was found that multiple cytoskeleton-associated proteins were among the core AEPs, which is consistent with the currently recognized phenomenon that the tubulin family controls organ asymmetry in many organisms by regulating cilia development^47^. Previous multivariate GWAS analyses have also found that functional annotations of gene loci associated with anatomical asymmetry in the adult brain include genes related to microtubules and prenatal brain development^15^. Protein expression patterns in the frontal lobe differed between hemispheres with increasing gestational age, with the expression of a cluster of proteins showing a continuous upwards trend on the left side but not on the right side.

The embryonic period ends at GW 8, when the basic structure of the brain and central nervous system has been established and the main sectors of the central and peripheral nervous systems have been identified. Neurons destined to form the neocortex, i.e., neuroblasts, are born beginning after the formation of the neural tube at GW 5, and the peak period of proliferation is from GW 6 to GW 18^48,49^. At GWs 12–20, these neurons migrate along a scaffolding formed by glial cells^50^. The early changes in protein expression in the frontal lobe may be due to cell proliferation and differentiation. The fetal stage of human development, which is the key period for the development of the neocortex^51^, begins at GW 9, and brain development mainly involves the production, migration and differentiation of neurons. According to our study, many DEPs between GWs 9–11 and GWs 11–13 are closely associated with neurogenesis.

We identified some proteins that showed asymmetric expression in the fetal frontal lobes. The inherent asymmetry of the human brain is important for brain function. Abnormal structural asymmetry and asymmetric activity of the human frontal lobe are related to many neuropsychiatric diseases, such as depression^52^, autism spectrum disorders^53^, and bipolar disorder^54^. Thus, the asymmetry of frontal lobe protein expression before birth, especially in the late first trimester and in the early second trimester, warrants intensive study, as it will help us to understand the asymmetry determined by genetics. Unlike the DEPs that were identified between different developmental time points, which were functionally similar, the AEPs identified between the left and right frontal lobes showed more subtle differences in both levels and associated biological processes. We confirmed that the pattern of protein expression changes in the frontal lobe was different between the two hemispheres. In the enrichment analysis of AEPs and proteins that may be expressed only unilaterally, we found that asymmetric expression of proteins was associated with processes related to nuclear export, RNA localization, RNA transport, organelle organization, extracellular matrix, adhesion and binding, and other biological processes. Notably, a previous study found that since the human brain is a tissue with complex cellular structures, global differences in organelle density or volume may confound explanations regarding differences in protein expression^25^. More research is needed to draw accurate conclusions about the causes of protein expression asymmetry. The development of brain asymmetry is more refined and is likely not determined by one or a few regulatory pathways. Furthermore, we believe that some proteins that are not significantly differentially expressed between the left and right brain may also play an indispensable role in the development of inherent asymmetry.

Due to ethical limitations, it is difficult to completely preserve the morphology of the fetus during abortion. Samples that can be used for preliminary proteomics research are very precious. Due to this limitation of a small sample size, we excluded proteins that were not consistently detected during the investigation of protein changes during development. Although imperfect, our study includes the very early age group of human brain tissue to be investigated for asymmetric protein expression, which can provide some clues for future research.

As the brain is the most complex organ in the human body, the difference in protein expression between the left and right brain deserves further attention. More research on different brain regions, changes in protein expression at shorter intervals and single-cell proteomics will be important for elucidating the process of fetal brain development and the genetic mechanism of brain asymmetry. We propose that the two sides of the brain should be analyzed separately in future multiomics research and human brain mapping studies. We will continue to pay attention to this issue in the future.

## Methods

### Ethical approval and consent to participate

Brain tissue was collected from foetuses discarded following induced pregnancy termination at Qilu Hospital, usually within 2 h of the procedure. The medical staff involved in conducting the pregnancy termination procedures were not involved in this scientific study. The Ethics Committee of Shandong University School of Basic Medical Sciences approved the study, and all participants provided written informed consent. All clinical information was collected, and the donors had no psychiatric disorders or a family history of psychiatric disorders. Ultrasonography was used to confirm that the fetal samples did not exhibit intracranial pathology.

### Sample preparation, protein expression quantification, and SDS–PAGE

Discarded brain tissues from three foetuses, one each at GWs 9, 11, and 13, were collected. The whole brains were harvested, and the left and right cerebral hemispheres were separated. Each hemisphere was dissected immediately with a scalpel, and tissues from the front part of the frontal lobes were collected (Fig. 1b). The samples were prepared for label-free experiments using the SDT lysis method. An appropriate amount of SDT lysis buffer (4% SDS, 100 mM Tris-HCl, pH 7.6) was added to the human fetal frontal lobe tissues, and the samples were transferred to Lysing Matrix A tubes and homogenized twice using a FastPrep-24 homogenizer (MP Biomedicals) (24 × 2, 6.0 m/s, 60 s) twice^55^. The homogenates were then placed in a boiling water bath for 10 min and centrifuged at 14,000 × *g* for 15 min. After centrifugation, the supernatants were collected and filtered through 0.22 μm Spin-X centrifuge tube filters. The protein concentration was quantified using the BCA method^56^ (BCA Protein Assay Kit, Beyotime), and 20 μg of each protein sample was subjected to 12% SDS–PAGE at 220 V for 40 min. The protein extracts were mixed with 6x sample buffer (Beyotime, P0015F) and put into a boiling water bath for 5 min. Coomassie Brilliant Blue was used for staining.

### FASP digestion

DTT was added at a final concentration of 100 mM to 200 μL of each protein solution^57^, and the samples were placed in a boiling water bath for 5 min and then cooled to room temperature. Then, 200 μL of UA buffer (8 M urea, 150 mM Tris-HCl pH 8.5) was added, the samples were transferred to 30 kD ultrafiltration centrifuge tubes and centrifuged at 12,500 × *g* for 15 min, and the filtrates were discarded. Then, this process was repeated once. Next, 100 μL IAA (100 mM IAA in UA) was added, and the samples were shaken at 600 rpm for 1 min, incubated for 30 min at room temperature in the dark and centrifuged at 12,500 × *g* for 15 min. Then, 100 μL UA buffer was added, and the samples were centrifuged at 12,500 × *g* for 15 min at room temperature. This process was repeated twice. After that, the samples were treated 100 μL 40 mM NH4HCO3 solution and centrifuged at 12,500 × *g* for 15 min at room temperature, and this process was repeated two times. A 40 μL aliquot of trypsin buffer (4 μg trypsin in 40 μL 40 mM NH4HCO3 buffer) was added, and then the samples were shaken at 600 rpm for 1 min and incubated at 37 °C for 16–18 h. Next, the samples were centrifuged at 12,500 × *g* for 15 min, treated with 20 μL of 40 mM NH4HCO3 buffer, and centrifuged at 12,500 × *g* for 15 min, and the filtrate was collected. The peptides were desalted with a C18 cartridge (Thermo Fisher Scientific), the peptide fragments were lyophilized and reconstituted, and the peptide concentration was quantified (OD280).

### LC–MS/MS analysis

The samples were separated by nanoflow liquid chromatography (Easy nLC, Thermo Fischer Scientific). Buffer A consisted of 0.1% formic acid in water, and buffer B consisted of 0.1% formic acid in 80% acetonitrile. The chromatographic column was equilibrated at 100% A, and LC separation was performed using a 50 µm × 15 cm Acclaim PepMap RSLC nano Viper column (Thermo Fisher Scientific) at a flow rate of 300 nL/min. The peptide fragments were separated by chromatography and analyzed by a Q-Exactive Plus mass spectrometer (Thermo Fisher Scientific). The scanning range of the parent ions was 350–1800 m/z, the resolution of first-order mass spectrometry was 70,000, the automatic gain control target was 3e6, and the first-order maximum IT was 50 ms. The mass-to-charge ratios of polypeptides and polypeptide fragments were obtained as follows. Twenty fragment patterns (MS2 scan, HCD) were collected after each full scan. The MS2 activation type was HCD, the isolation window was 2 m/z, the resolution of secondary mass spectrometry was 17,500, the microscan was 1, the secondary maximum IT was 45 ms, and the normalized collision energy was set to 27 eV.

### Protein identification and quantification

Raw data were searched against the Uniprot_HomoSapiens_20377_20220308 database. Protein identification and quantification were performed in MaxQuant^58^ version 2.1.1.0 (Max Planck Institute of Biochemistry) in a single run using the following parameters^34^: the enzyme was set to trypsin, two missed cleavages were allowed, the fixed modifications were set to carbamidomethyl (C), the variable modifications were set to oxidation (M) and acetyl (Protein N-term), and the first and main search mass tolerances were set to 20 and 4.5 parts per million (ppm), respectively. A common contamination database was included to eliminate the effect of contamination proteins among the identified proteins. The identified peptides and proteins were filtered at a FDR of 0.01. The protein abundance was calculated based on the normalized spectral protein intensity (LFQ intensity). The LFQ minimum ratio count was set to two. The match between run option was activated.

### Screening of DEPs and AEPs

To visualize the proteins that were identified on only one side of the frontal lobe, an UpSetR diagram was created using the R package UpsetR v1.4.0^59^. Venn diagrams were generated using the online tool jvenn^60^.

Proteins that were detected in the bilateral frontal lobes in all samples were identified as the core protein set. The R package NormalyzerDE^61^ was used to normalize the abundance of the core proteins, and log2 normalization was applied to identify the DEPs between different GWs. In the study of proteins differentially expressed during development, we used limma differential expression analysis^62^ to determine protein expression differences among gestational weeks and considered the frontal lobe as a whole, using a fold change >2 and FDR < 0.05 as the criteria. The result of limma differential expression analysis and the list of the DEPs can be found in Supplementary Data 3 and 4, respectively.

The proteins that were detected in the bilateral frontal lobes at each GW were used for AEPs screening. Proteins with a fold change in LFQ intensity >2 and significance A value <0.05 between the left and right frontal lobes were considered to be asymmetrically expressed. In the significance A algorithm, the normal distribution was arranged according to the expression of the two groups of samples, and the *p* value was calculated according to the significance ratio score^58^. The fold change data for the AEPs were used to generate a heatmap using TBtools software^63^. Values were scaled and clustered by row.

### PPI network analysis

PPI networks are of great significance for revealing the interactions between proteins. In this study, the online tool Search Tool for the Retrieval of Interacting Genes/Proteins (STRING) (http://string-db.org) was used, with the STRING human database serving as a statistical background. For the functional enrichments in the network, the whole genome was used as the statistical background.

### Enrichment analysis, UniProt keyword annotation, and functional annotation clustering

GO enrichment analysis were performed with the R package “ClusterProfiler”^64^ and Bioconductor annotation package “org.Hs.eg.db”. The “GOplot” package was used to visualize the distribution of AEPs enriched in certain GO terms^65^. UniProt keyword annotation was performed using UniProt Retrieve/ID mapping (https://www.uniprot.org/). Functional annotation clustering was carried out using DAVID (https://david.ncifcrf.gov/) with the default *Homo sapiens* genome as the background.

### STEM analysis

In the current study, we used STEM software^66^ to study the change in the expression pattern of core proteins in the bilateral frontal lobes with age. The log2 normalized LFQ values of the core proteins were subjected to downstream clustering analysis based using the default settings. Clusters with a *p* value ≤ 0.05 were considered statistically significant.

### Statistics and reproducibility

Correlation analysis and PCA were carried out with the cor and prcomp functions, respectively, of R using the correlation matrix of the log2 normalized core protein data. These proteins were repeatedly quantified in six frontal lobes of all three samples. The PCA plot was generated using ggplot2 (version 3.3.5) and ggalt (version 0.4.0). The correlation plot was generated using ggcorrplot (version 0.1.3).

### Reporting summary

Further information on research design is available in the Nature Research Reporting Summary linked to this article.

## Supplementary information

Supplementary Information

Description of Additional Supplementary Files

Supplementary Data 1

Supplementary Data 2

Supplementary Data 3

Supplementary Data 4

Supplementary Data 5

Supplementary Data 6

Supplementary Data 7

Supplementary Data 8

Supplementary Data 9

Supplementary Data 10

Supplementary Data 11

Supplementary Data 12

Reporting Summary

## Data Availability

The mass spectrometry proteomics data generated and analyzed in this study have been deposited to the ProteomeXchange with the dataset identifier PXD034192. All data sets used for figures are provided in the supplementary data 12.
