# Supplementary material for: Changes in and asymmetry of the proteome in the human fetal frontal lobe during early development
Source: Commun Biol. 2022 Sep 29;5:1031. doi: 10.1038/s42003-022-04003-6 (PMC9522861; doi:10.1038/s42003-022-04003-6)
Supplement: Supplementary file 1 — Supplementary Information [file 42003_2022_4003_MOESM1_ESM.pdf]

Supplementary Figure 1. GO enrichment of 2281 core proteins that detected in six tissues

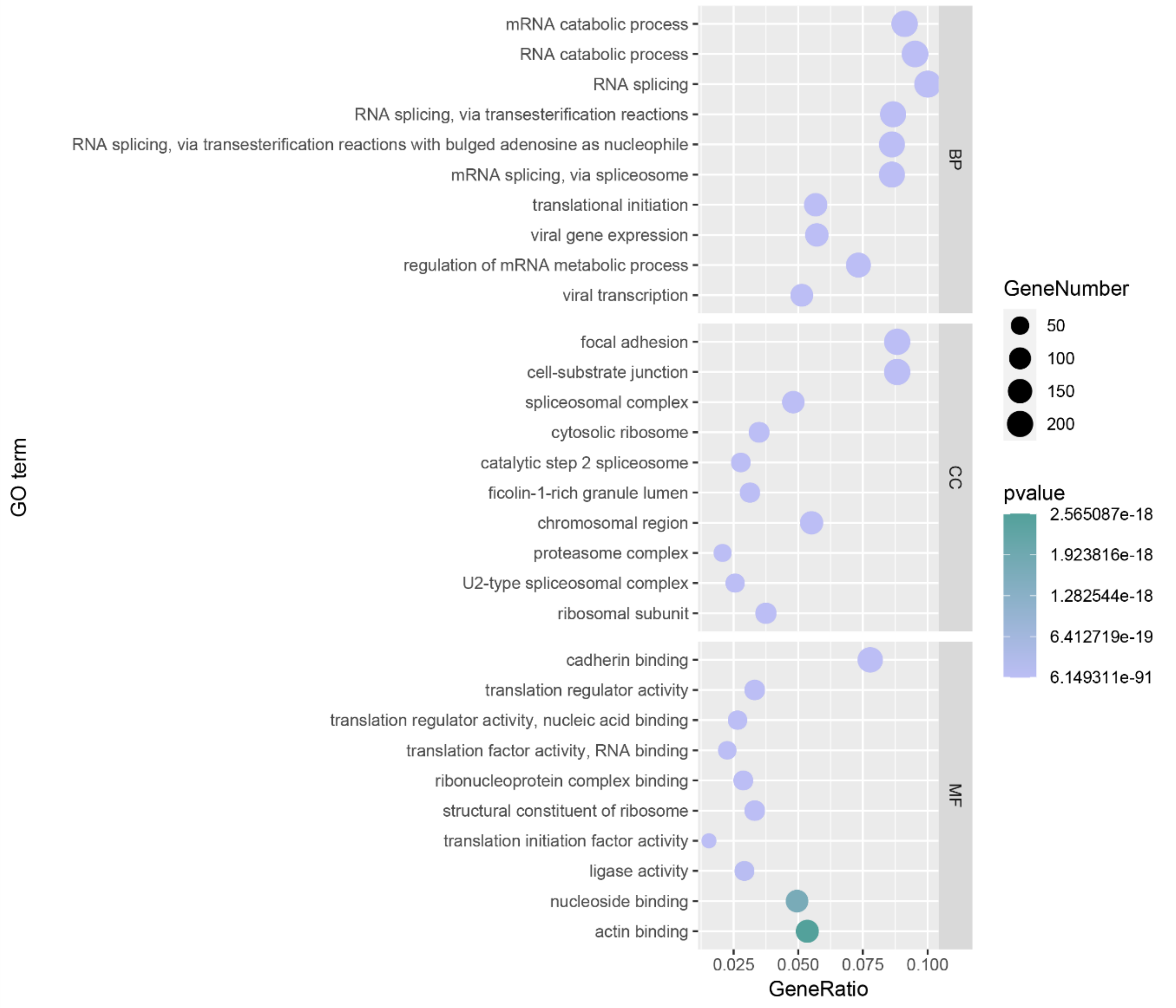

The X axis shows GeneRatio, the Y axis shows the enriched GO terms. The Dot size indicates the numbers of proteins found in each enriched GO term; the dot color indicates the adjusted p-value.

Supplementary Figure 2. Scatter plot of averaged expression plotted against asymmetry index

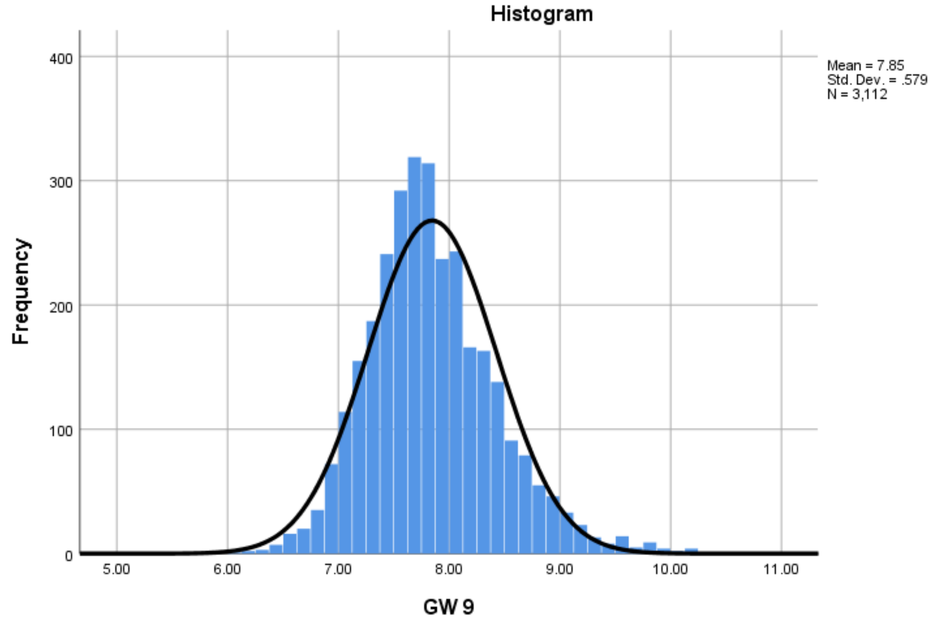

a

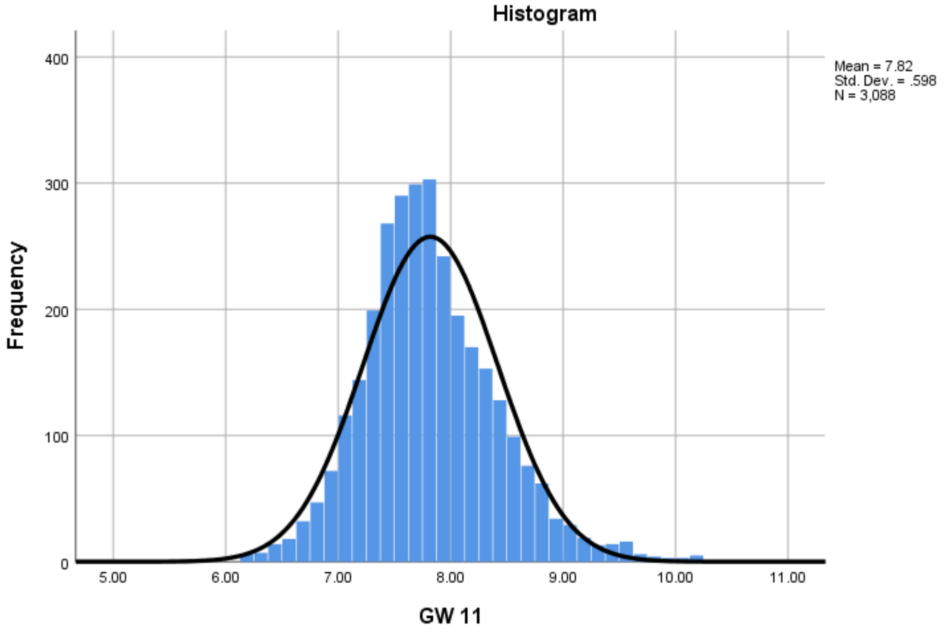

b

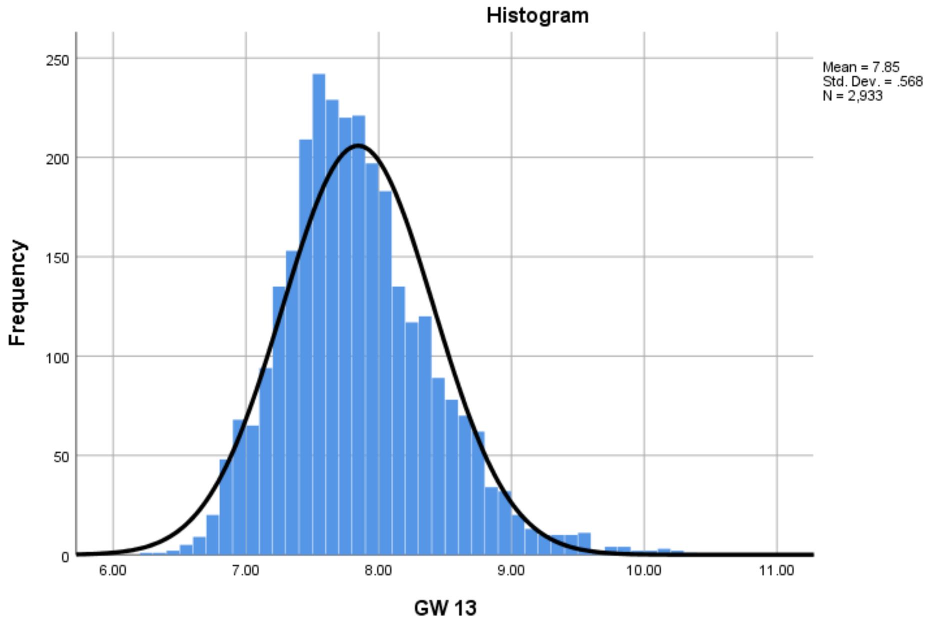

c

		GW9	GW 11	GW 13
N		3112	3088	2933
Mean		7.8465	7.8196	7.8454
Median		7.7924	7.7594	7.7835
Std. Deviation		.57914	.59791	.56829
Skewness		.575	.542	.666
Std. Error of Skewness		.044	.044	.045
Kurtosis		.874	.918	.919
Std. Error of Kurtosis		.088	.088	.090
Percentiles	1	6.6480	6.5198	6.7663
	99	9.5413	9.5391	9.5236

a. Multiple modes exist. The smallest value is shown

d

a-c Histograms and normal curves of average protein intensity in GW 9, 11 and 13.
d Descriptions of the mean, median, standard deviation, skewness, kurtosis, standard error skewness and kurtosis, and the 1st , 99th percentiles.
