# Supplementary material for: Changes in and asymmetry of the proteome in the human fetal frontal lobe during early development
Source: Commun Biol. 2022 Sep 29;5:1031. doi: 10.1038/s42003-022-04003-6 (PMC9522861; doi:10.1038/s42003-022-04003-6)
Supplement: Supplementary file 2 — Description of Additional Supplementary Files [file 42003_2022_4003_MOESM2_ESM.pdf]

Description of Additional Supplementary Files

File name: Supplementary Data 1

Description: Protein identification list of GWs 9, 11 and 13.

File name: Supplementary Data 2

Description: GO enrichment analysis of 2281 core proteins detected in all six tissues. Table of GO enrichments using the Fisher exact test (Benjamini-Hochberg corrected p-values are shown).

File name: Supplementary Data 3

Description: Limma result of differentially expressed proteins.

File name: Supplementary Data 4

Description: List of significant differentially expressed proteins between 9-11 GW and 11-13 GW.

File name: Supplementary Data 5

Description: List of asymmetrically expressed proteins at GWs 9, 11, and 13.

File name: Supplementary Data 6

Description: List of 2-fold change asymmetrically expressed proteins shared between at least two gestational weeks.

File name: Supplementary Data 7

Description: List of 1.5-fold change asymmetrically expressed proteins shared between at three gestational weeks.

File name: Supplementary Data 8

Description: GO enrichment analysis of 2-fold change asymmetrically expressed proteins in GWs 9, 11, and 13. Table of GO enrichments using the Fisher exact test (Benjamini-Hochberg corrected p-values are shown).

File name: Supplementary Data 9

Description: List of proteins detected only on the left and right frontal lobe.

File name: Supplementary Data 10

Description: List of proteins significantly enriched by STEM analysis.

File name: Supplementary Data 11

Description: Functional annotation clustering analysis of profile 4 in the right frontal lobe.

File name: Supplementary Data 12

Description: All data sets used for figures.
