# Supplementary material for: Changes in and asymmetry of the proteome in the human fetal frontal lobe during early development
Source: Commun Biol. 2022 Sep 29;5:1031. doi: 10.1038/s42003-022-04003-6 (PMC9522861; doi:10.1038/s42003-022-04003-6)
Supplement: Supplementary file 15 — Reporting Summary [file 42003_2022_4003_MOESM15_ESM.pdf]

nature research

Corresponding author(s): Shuwei Liu, Yuchun Tang

Last updated by author(s): Sep 8, 2022

Reporting Summary

Nature Research wishes to improve the reproducibility of the work that we publish. This form provides structure for consistency and transparency in reporting. For further information on Nature Research policies, see our Editorial Policies and the Editorial Policy Checklist.

Statistics

For all statistical analyses, confirm that the following items are present in the figure legend, table legend, main text, or Methods section.

n/a	Confirmed	
☐	☒	The exact sample size (*n*) for each experimental group/condition, given as a discrete number and unit of measurement
☐	☒	A statement on whether measurements were taken from distinct samples or whether the same sample was measured repeatedly
☐	☒	The statistical test(s) used AND whether they are one- or two-sided<br>*Only common tests should be described solely by name; describe more complex techniques in the Methods section.*
☒	☐	A description of all covariates tested
☒	☐	A description of any assumptions or corrections, such as tests of normality and adjustment for multiple comparisons
☐	☒	A full description of the statistical parameters including central tendency (e.g. means) or other basic estimates (e.g. regression coefficient) AND variation (e.g. standard deviation) or associated estimates of uncertainty (e.g. confidence intervals)
☒	☐	For null hypothesis testing, the test statistic (e.g. *F*, *t*, *r*) with confidence intervals, effect sizes, degrees of freedom and *P* value noted<br>*Give P values as exact values whenever suitable.*
☒	☐	For Bayesian analysis, information on the choice of priors and Markov chain Monte Carlo settings
☒	☐	For hierarchical and complex designs, identification of the appropriate level for tests and full reporting of outcomes
☐	☒	Estimates of effect sizes (e.g. Cohen's *d*, Pearson's *r*), indicating how they were calculated

Our web collection on statistics for biologists contains articles on many of the points above.

Software and code

Policy information about availability of computer code

Data collection	The samples were separated by nanoflow liquid chromatography (Easy nLC, Thermo Fischer Scientific).<br>The peptide fragments were separated by chromatography and analysed by a Q-Exactive Plus mass spectrometer (Thermo Fisher Scientific).
Data analysis	Protein identification and quantification were performed in MaxQuant version 2.1.1.0 (Max Planck Institute of Biochemistry).<br>UpSetR diagram was created using the R package UpsetR v1.4.0.<br>The R package NormalyzerDE was used to normalize the abundance of the core proteins.<br>The fold change data for the AEPs were used to generate a heatmap using TBtools software.<br>PPI networks were analyzed by the online tool Search Tool for the Retrieval of Interacting Genes/Proteins (STRING).<br>GO enrichment analysis and GSEA were performed with the R package 'ClusterProfiler' and Bioconductor annotation package 'org.Hs.eg.db'.<br>Functional annotation clustering was carried out using DAVID (https://david.ncifcrf.gov/).<br>STEM software was used to study the change in the expression pattern of core proteins in the bilateral frontal lobes with age.<br>Correlation analysis and PCA were carried out with the cor and prcomp functions of R.

For manuscripts utilizing custom algorithms or software that are central to the research but not yet described in published literature, software must be made available to editors and reviewers. We strongly encourage code deposition in a community repository (e.g. GitHub). See the Nature Research guidelines for submitting code & software for further information.

Data

Policy information about availability of data

All manuscripts must include a data availability statement. This statement should provide the following information, where applicable:

- Accession codes, unique identifiers, or web links for publicly available datasets
- A list of figures that have associated raw data
- A description of any restrictions on data availability

The mass spectrometry proteomics data generated and analysed in this study have been deposited to the ProteomeXchange with the dataset identifier PXD034192. Other data supporting the findings of this study are available within the article and its supplementary data files.

Field-specific reporting

Please select the one below that is the best fit for your research. If you are not sure, read the appropriate sections before making your selection.

☒ Life sciences ☐ Behavioural & social sciences ☐ Ecological, evolutionary & environmental sciences

For a reference copy of the document with all sections, see nature.com/documents/nr-reporting-summary-flat.pdf

Life sciences study design

All studies must disclose on these points even when the disclosure is negative.

Sample size	The number of specimens for proteomics was three.
Data exclusions	No data were excluded from the analysis.
Replication	A total of 2650 proteins were identified in all three samples, 85.2 - 90.4% of all proteins identified in each sample. Proteins quantified in all six frontal lobes of three samples were used for differential analysis between gestational weeks.
Randomization	This is not relevant to our study because our studies has only one sample in each gestational week.
Blinding	The medical staff involved in conducting the pregnancy termination procedures were not involved in this scientific study.

Reporting for specific materials, systems and methods

We require information from authors about some types of materials, experimental systems and methods used in many studies. Here, indicate whether each material, system or method listed is relevant to your study. If you are not sure if a list item applies to your research, read the appropriate section before selecting a response.

Materials & experimental systems

n/a	Involved in the study	
☒	☐	Antibodies
☒	☐	Eukaryotic cell lines
☒	☐	Palaeontology and archaeology
☒	☐	Animals and other organisms
☒	☐	Human research participants
☒	☐	Clinical data
☒	☐	Dual use research of concern

Methods

n/a	Involved in the study	
☒	☐	ChIP-seq
☒	☐	Flow cytometry
☒	☐	MRI-based neuroimaging
